# High-Fat Diet Augments VPAC1 Receptor-Mediated PACAP Action on the Liver, Inducing LAR Expression and Insulin Resistance

**DOI:** 10.1155/2016/9321395

**Published:** 2016-12-01

**Authors:** Masanori Nakata, Boyang Zhang, Yifei Yang, Takashi Okada, Norihito Shintani, Hitoshi Hashimoto, Toshihiko Yada

**Affiliations:** ^1^Department of Physiology, Division of Integrative Physiology, Jichi Medical University School of Medicine, Shimotsuke, Tochigi 329-0498, Japan; ^2^Department of Biochemistry and Molecular Biology, Division of Gene Therapy, Research Center for Advanced Medical Technology, Nippon Medical School, Tokyo 113-8603, Japan; ^3^Laboratory of Molecular Neuropharmacology, Graduate School of Pharmaceutical Sciences, Osaka University, Suita, Osaka 565-0871, Japan; ^4^Molecular Research Center for Children's Mental Development, United Graduate School of Child Development, Osaka University, Kanazawa University, Hamamatsu University School of Medicine, Chiba University and University of Fukui, Suita, Osaka 565-0871, Japan; ^5^Division of Bioscience, Institute for Datability Science, Osaka University, Suita, Osaka 565-0871, Japan

## Abstract

Pituitary adenylate cyclase-activating polypeptide (PACAP) acts on multiple processes of glucose and energy metabolism. PACAP potentiates insulin action in adipocytes and insulin release from pancreatic *β*-cells, thereby enhancing glucose tolerance. Contrary to these effects at organ levels, PACAP null mice exhibit hypersensitivity to insulin. However, this apparent discrepancy remains to be solved. We aimed to clarify the mechanism underlying the antidiabetic phenotype of PACAP null mice. Feeding with high-fat diet (HFD) impaired insulin sensitivity and glucose tolerance in wild type mice, whereas these changes were prevented in PACAP null mice. HFD also impaired insulin-induced Akt phosphorylation in the liver in wild type mice, but not in PACAP null mice. Using GeneFishing method, HFD increased the leukocyte common antigen-related (LAR) protein tyrosine phosphatase in the liver in wild type mice. Silencing of LAR restored the insulin signaling in the liver of HFD mice. Moreover, the increased LAR expression by HFD was prevented in PACAP null mice. HFD increased the expression of VPAC1 receptor (VPAC1-R), one of three PACAP receptors, in the liver of wild type mice. These data indicate that PACAP-VPAC1-R signaling induces LAR expression and insulin resistance in the liver of HFD mice. Antagonism of VPAC1-R may prevent progression of HFD-induced insulin resistance in the liver, providing a novel antidiabetic strategy.

## 1. Introduction

Pituitary adenylate cyclase-activating polypeptide (PACAP) regulates glucose and energy metabolism through central and peripheral actions. PACAP potentiates glucose-induced insulin release from pancreatic islet and promotes insulin-mediated glucose disposal in adipocyte [1–3]. In addition, in hunger and fasted conditions, PACAP increases food intake and catecholamine release to maintain the energy supply [4–6]. PACAP is localized in the central and peripheral tissues [7]. In the central nervous system (CNS), PACAP is localized abundantly in the hypothalamus [8] and in the brain stem, including the nucleus tractus solitarius (NTS) and dorsal motor vagal nucleus (DMV) [9], the areas that regulate energy metabolism. PACAP is also localized in a wide range of peripheral nerves including the sympathetic nervous system. Thus, PACAP is thought to act as a neurotransmitter or neuromodulator in the central and peripheral nerves.

There are three types of PACAP receptors: PAC1 receptor (PAC1-R) selective for PACAP [10], and VPAC1-R and VPAC2-R shared by PACAP and VIP with equal potencies [11]. Among three types of PACAP receptors, PAC1-R is expressed in pancreatic islets and VPAC2-R in pancreatic islets and adipocyte [12, 13]. PACAP enhances glucose-stimulated insulin secretion in islets and insulin-induced glucose uptake in adipocytes, the effects acting as antidiabetic [1–3]. Contradictory to these actions, PACAP null mice reportedly exhibit increased insulin sensitivity with regular chow diet [14]. Moreover, PACAP null mice are resistant against high-fat diet- (HFD-) induced insulin resistance. These results indicate that PACAP might impair the insulin action in the tissues other than adipose tissue. The liver is one of the major organs that are targeted by insulin and implicated in insulin resistance, and it expresses PACAP receptors. It was previously reported that PACAP reportedly stimulates adenylate cyclase in hepatocytes and glucose output from them [15, 16]. Hence, the liver could be the candidate target for the PACAP action on glucose metabolism. In this study, we aimed to elucidate the signal mechanism and function of PACAP in the liver of mice fed HFD.

## 2. Materials and Methods

### 2.1. PACAP Null Mice and Wild Type Mice

The generation and maintenance of the PACAP null mice on the ICR background have been previously described in detail [4]. Five-week-old male ICR mice and C57BL/6J mice were obtained from Japan SLC (Hamamatsu, Japan). In this study, PACAP null mice and wild type ICR mice were single-housed under a 12 h light : 12 h dark cycle (lights on at 07:30 a.m.) conditions. Mice were randomly divided into two groups fed with either a regular chow or a HFD for 6 weeks. In the chow (CE-2; CLEA, Japan), 58.9% of calories was derived from carbohydrate and 11.6% of calories was derived from fat, yielding total calories of 344 kcal/100 g. In the HFD (Quick fat CLEA-Japan, Tokyo, Japan), 44.6% of calories was derived from carbohydrate containing sucrose and 32.3% of calories was derived from fat, 425 kcal/100 g. Experimental procedures and care of animals were carried out according to the Jichi Medical University Institute of Animal Care and Use Committee.

### 2.2. Glucose Tolerance and Insulin Tolerance Tests

For glucose tolerance tests (GTT), mice at 6 weeks after regular chow and HFD fed were fasted 6-h. Following administration of glucose (2 g per kg i.p.), glucose levels were measured in tail blood at indicated time points. For insulin tolerance tests (ITT), mice were fasted 6-h. Following administration of insulin (0.5 IU per kg i.p.), glucose levels were measured in tail blood at indicated time points.

### 2.3. GeneFishing Analysis of Mouse Liver RNA Expression

C57BL/6J mice at six weeks old were randomly assigned to one of two different dietary groups for 6 weeks (*n* = 4). At 6 weeks after regular chow or HFD fed, total RNA of liver was isolated using TRIzol (Invitrogen, Carlsbad, CA) and treated with RQ1-DNase (Promega, Madison, WI) to remove residual contaminations with DNA. To analyze the difference of RNA expression of both group mice liver, we used a new differential display PCRs method that is based on annealing control primers (ACPs). Reverse transcription was conducted using the GeneFishing™ DEG kits (Seegene, Seoul, Korea) as follows; 3 *μ*g of total RNA was converted to cDNA using ReverTra Ace (Toyobo, Osaka, Japan) with dT-ACP1 primer. The synthesized first-strand cDNA samples were subjected to PCR amplification using dT-ACP2 and one of arbitrary ACPs as primer. After incubation at 94°C for 5 min, 50°C for 3 min, and 72°C for 1 min, 40 cycles of 94°C for 40 sec, 65°C for 40 sec, and 72°C for 40 sec followed, after which a postextension was performed at 72°C for 5 min. The PCR products were then subjected to electrophoresis on a 2% agarose gel and stained with 1 mg/mL ethidium bromide. Amplified PCR products were cloned into a pGEM-T vector using the TA cloning system (Takara Bio, Ohtsu, Japan) for further analysis. Double-stranded DNA was sequenced by Hitachi model SQ-5500 DNA auto-sequencer by using delta Taq fluorescent dye-primer cycle sequencing kit (Amersham Corp., Arlington Heights, IL) and Texas-red labelled primer.

### 2.4. Construction of Short Hairpin RNAs (shRNAs) and Viral Vector Production

Target sequence for mouse LAR was chosen to design shRNAs (accession number NM 011213.2: 5′-CGAATTACGTGGATGAAGA-3′. 530 to 548). In addition, scrambled oligonucleotide sequence was used for specificity control (5′-CAACACTAGTTGACATGTA-3′). The mU6 promoter, hairpin sequence and terminator sequences were subcloned into pAAV plasmid. The shRNA constructs were then cloned into an AAV serotype 9 (AAV9) for stable shRNA delivery. Briefly, AAV9-LAR-shRNA and AAV9-Scr-shRNA viruses were produced following triple-transfection of HEK293 cells with pAAV-shRNA, an adenoviral helper plasmid pAdeno, and a chimeric helper plasmid encoding AAV2 rep/AAV9 cap genes (pAAV2rep/AAV9cap). The purification and titration of AAV were reported as previously described [17].

### 2.5. Administration of AAV Vectors In Vivo

For intraliver administration, vectors were dissolved in 100 *μ*L saline and injected directly into the portal vein of the mice. AAV-LAR-shRNA or control AAV-scrambled-shRNA (1 × 10^12^ vg) was administered to 6 weeks HFD fed C57BL/6 mice. At 1 weeks after administration of AAV vectors, insulin tolerance test and measurement of the insulin receptor phosphorylation in liver of both group mice were performed.

### 2.6. Western Blot

Phosphorylation of Akt and insulin receptor was measured by western blot as previously reported [18]. After intraperitoneal administration of insulin (0.5 IU per kg i.p.), livers were removed and lysed in 1 mL lysis buffer containing 100 mM sodium fluoride, 10 mM sodium PPi, and 2 mM sodium orthovanadate. The 10 *μ*g proteins were subjected to 8% SDS-PAGE and transferred to nitrocellulose filters. Phosphorylation of Akt and insulin receptor were detected with the polyclonal antibodies, phospho-Ser473 Akt (CST, Beverly, MA), or phosphor-Tyr1361 insulin receptor *β*-subunit (Abcam plc, Cambridge, UK). Immunoreactive proteins were detected with HRP-conjugated secondary antibody and the ECL system (Amersham Corp., Arlington Heights, IL). After stripping, membrane was hybridized with anti-Akt antibody (CST, Beverly, MA) or anti-insulin receptor *β*-subunit (Santa Cruz bio., Santa Cruz, CA). Then, immunoreactive signal was quantified by FAS-1000 (Fujifilm, Japan) and signal levels of phosphor-Akt and phosphor-insulin receptor were normalized to Akt and insulin receptor *β*-subunit.

Liver lysate proteins of mice administrated of AAV vectors were subjected to 8% SDS-polyacrylamide gel electrophoresis and transferred to nitrocellulose filters. LAR proteins were detected with the anti-LAR IgG (Transduction Lab, Lexington, KY) and ECL system. Immunoreactive signal was quantified by using FAS-1000 (Fujifilm, Tokyo, Japan) and expression levels of proteins were normalized to *β*-actin (Santa Cruz bio.).

### 2.7. Measurements of mRNA

Quantification of mRNA expressions measured by real-time PCR has been previously described in detail [19]. Total RNA was isolated using Trizol and RNase-free DNase. After the conversion to cDNA, real-time PCR was performed with SYBR premix Ex taq II polymerase (Takara Bio). Expression levels were calculated by the ΔΔCT method. The primers used in this experiment were listed in Supplemental Table  1 in Supplementary Material available online at http://dx.doi.org/10.1155/2016/9321395.

### 2.8. Liver Slice Culture

Transverse liver slices were prepared from 5-week-old male ICR mice as previously reported [20]. Liver was isolated from anesthetized mice, from which slices of 300 *μ*m thickness were prepared with a vibratome. Tissue slices were explanted on a culture membrane (Millicell CM, pore size 0.4 *μ*m, Millipore) in a 35 mm Petridish sealed with Parafilm (American Can Co., Greenwich) and cultured with test reagents in 1.0 mL DMEM (Gibco-Invitrogen), which was supplemented with 2.7 mM NaHCO_3_, 10 mM HEPES, 20 mg/L kanamycin (Gibco), 100 mg/mL apo-transferin (Sigma), 5 *μ*g/mL insulin (Sigma), 100 mm putrescine (Sigma), 20 nM progesterone (Sigma), and 30 nM sodium selenite (Gibco) and incubated in 5% CO_2_ at 37°C for overnight. After incubation for 24 hours, total RNAs were extracted and RT-PCR was performed as described earlier.

### 2.9. Statistical Analysis

All data are expressed as mean ± sem. All data were subjected to one-way or two-way ANOVA. Differences between experimental groups were determined by the Tukey test. Statistical significance was accepted when *p* < 0.05.

## 3. Results

### 3.1. PACAP Null Mice Were Protected against HFD-Induced Insulin Resistance

**Figure 1:**
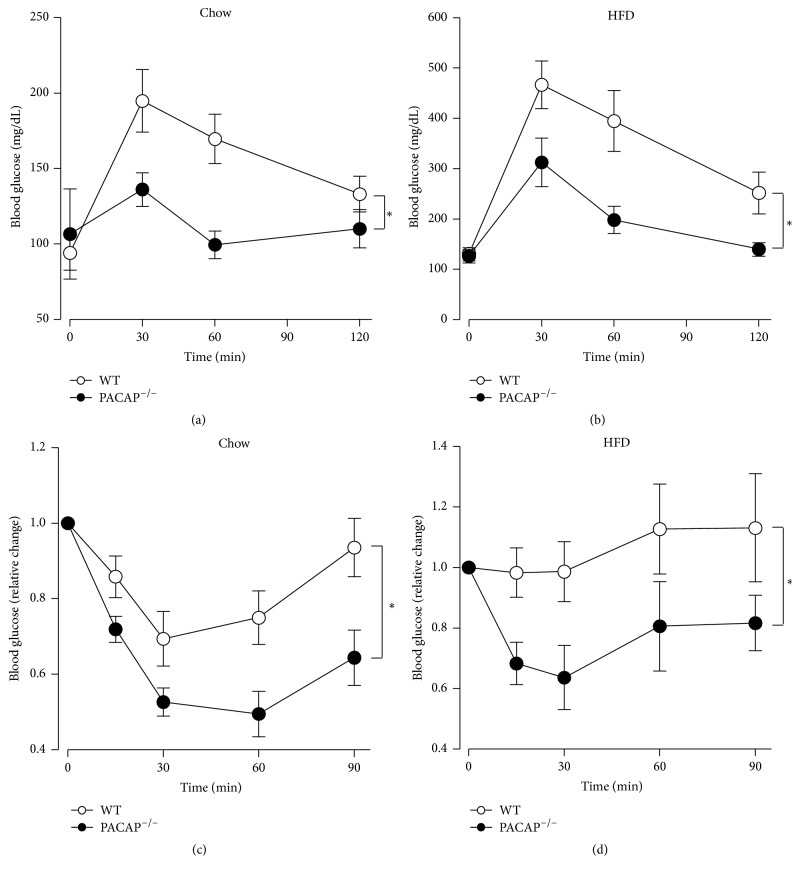
Effects of HFD on glucose tolerance and insulin tolerance in wildtype and PACAP null mice. (a) and (b) Blood glucose levels in GTT in wild type (WT) (open symbols) and PACAP null mice (filled symbols) fed regular chow (a) and HFD (b) for 6 weeks. Glucose at 2 g/kg body weight was injected to mice fasted 6 h. (c) and (d) Blood glucose levels in ITT in WT and PACAP null mice fed regular chow (c) and HFD (d) for 6 weeks. Insulin at 0.5 IU/kg body weight was injected to mice fasted for 6 h. All data are presented as means ± s.e.m. ^*∗*^ *p* < 0.05.

Mice fed regular chow or HFD for 6 weeks were subjected to GTTs. Under regular chow fed conditions, PACAP null mice, compared to wild type mice, exhibited a remarkably suppressed rise of blood glucose and quicker return to the basal level at 60 min (Figure 1(a)). Under HFD fed conditions, blood glucose was elevated to high levels over 400 mg/dL in wild type mice, which was markedly ameliorated in PACAP null mice (Figure 1(b)). In ITTs, under regular chow fed conditions, PACAP null mice exhibited the greater insulin sensitivity compared to wild type mice (Figure 1(c)). Furthermore, the severe insulin resistance induced by HFD was ameliorated in PACAP null mice (Figure 1(d)). These results indicate that PACAP deficiency promotes insulin sensitivity under regular chow fed conditions and counteracts insulin resistance induced by HFD.

**Figure 2:**
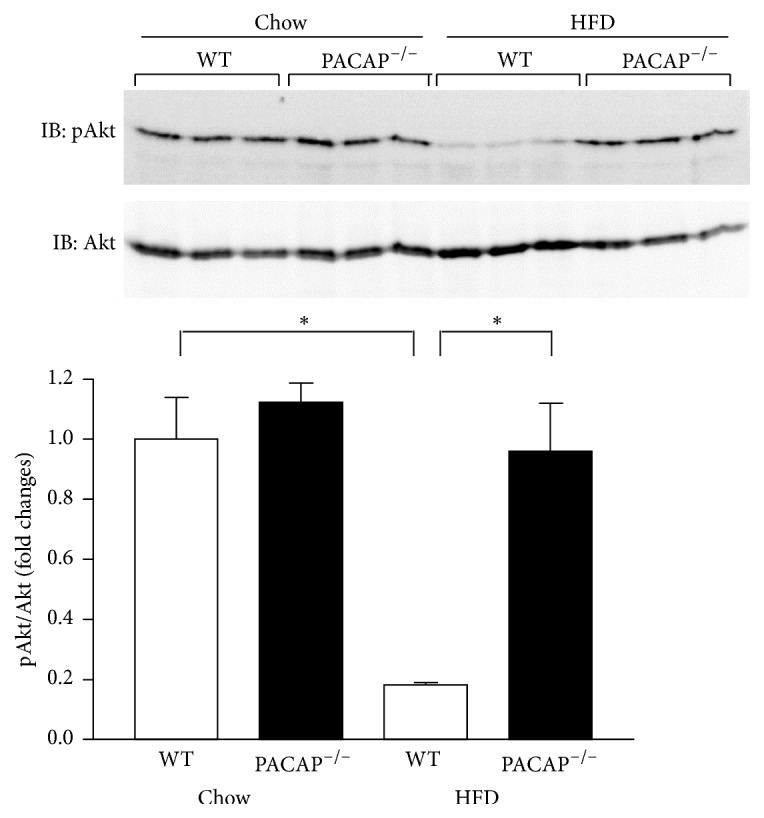
Insulin-induced Akt phosphorylation in liver. Western blot analysis using anti-phospho Akt and anti-Akt antibodies. Liver samples were collected form wild type (WT) and PACAP null (PACAP^−/−^) mice fed regular chow and HFD for 6 weeks. The intensity of Akt phosphorylation is expressed by the ratio of phosphorylated over total Akt proteins. *n* = 3. All data are presented as means ± s.e.m. ^*∗*^ *p* < 0.05.

To explore the mechanism for the enhanced insulin sensitivity in PACAP null mice, the phosphorylation (Ser473) of Akt, an insulin signaling molecule, in the liver was examined. Liver tissue samples were obtained from anaesthetized mice at 15 min after i.p. insulin injection (0.5 IU/kg body weight). Insulin was marked by phosphorylated Akt in the liver of wild type and PACAP null mice fed regular chow (Figure 2). Under HFD fed conditions, in contrast, Akt phosphorylation was markedly reduced in wild type mice, and this change was almost completely prevented in PACAP null mice, while total Akt protein content remained unchanged (Figure 2). These results indicate that PACAP counteracts the action of HFD to impair insulin-induced Akt phosphorylation in the liver.

### 3.2. The Expression of LAR in Liver Was Elevated in Mice Fed HFD

**Figure 3:**
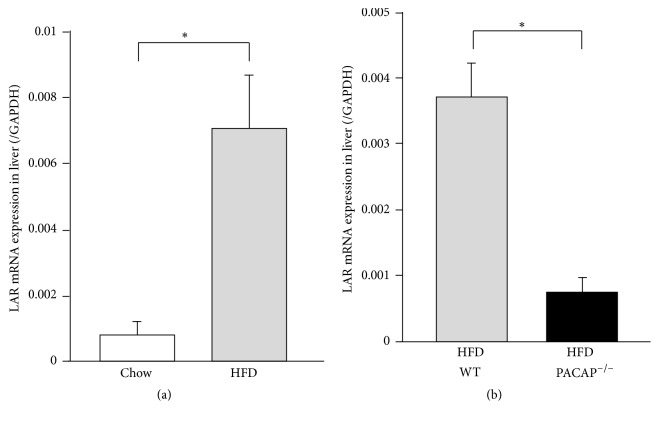
LAR expression in liver of mice. (a) LAR mRNA expression in the liver of regular chow fed and HFD fed ICR mice at 6 weeks. *n* = 5 to 6. (b) LAR mRNA expression in the liver of wild type and PACAP null mice fed HFD for 6 weeks. *n* = 5 to 6. All data are presented as means ± s.e.m. ^*∗*^ *p* < 0.05.

To explore the molecules induced by HFD, the differences in mRNA expression in the liver between regular chow and HFD conditions were analyzed using GeneFishing. In the liver of mice fed HFD, 21 elevated genes and 4 reduced genes were identified. One elevated gene was leukocyte common antigen-related (LAR) protein tyrosine phosphatase, also known as receptor type protein tyrosine phosphatase F (PTPRF), which is involved in insulin signaling [15]. Quantitative PCR also showed that HFD treatment markedly elevated LAR mRNA expression in the liver (Figure 3(a)). On the other hand, the elevated LAR mRNA expression in the liver under HFD conditions was significantly decreased in PACAP null mice (Figure 3(b)), suggesting that PACAP increases LAR mRNA expression under HFD conditions.

**Figure 4:**
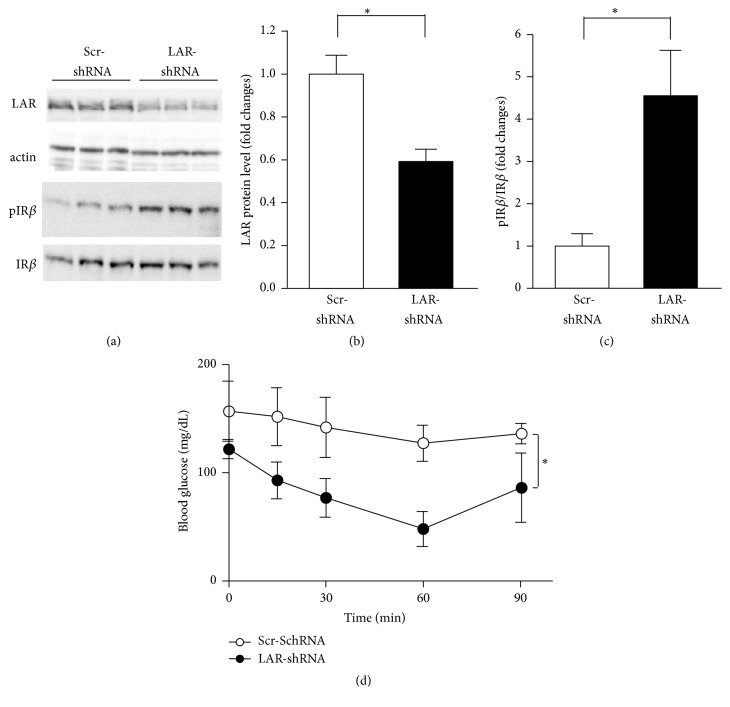
Effects of LAR knockdown on insulin sensitivity in liver of wild type mice fed HFD. (a) Western blot analysis using anti-LAR and anti-phospho-insulin receptor *β*-subunit (pIR*β*) in the liver of HFD mice at 1 week after injection of AAV-Scr-shRNA or LAR-shRNA. Liver samples were collected at 10 min after insulin injection (0.5 IU/kg). (b) LAR protein levels, relative to *β*-actin (actin), in the liver. *n* = 3 for each group. (c) The intensity of IR*β* phosphorylation is expressed by the ratio of phosphorylated over total insulin receptor *β*-subunit (IR*β*) proteins. (d) Blood glucose levels in ITT in HFD mice at 1 week after injection of AAV-Scr-shRNA or LAR-shRNA. Insulin at 0.5 IU/kg body weight was injected to mice fasted for 6 h. All data are presented as means ± s.e.m. ^*∗*^ *p* < 0.05.

To assess the pathological link between overexpression of LAR and insulin resistance in HFD mice, we silenced LAR by using shRNA expression AAV vector. Intraportal administration of AAV-LAR-shRNA vector significantly reduced the expression of LAR in liver of HFD mice (Figures 4(a) and 4(b)). The treatment with LAR-shRNA, compared to Scr-shRNA, ameliorated insulin resistance in HFD mice (Figures 4(a) and 4(c)). Furthermore, the phosphorylation of insulin receptor *β*-subunits in the liver in AAV-LAR-shRNA administrated mice was significantly increased compared to that of control mice. AAV-LAR-shRNA administration significantly improved insulin sensitivity in HFD mice (Figure 4(d)). These results indicated that the overexpression of LAR in liver, which was induced by HFD through PACAP, impaired the insulin sensitivity in vivo.

**Figure 5:**
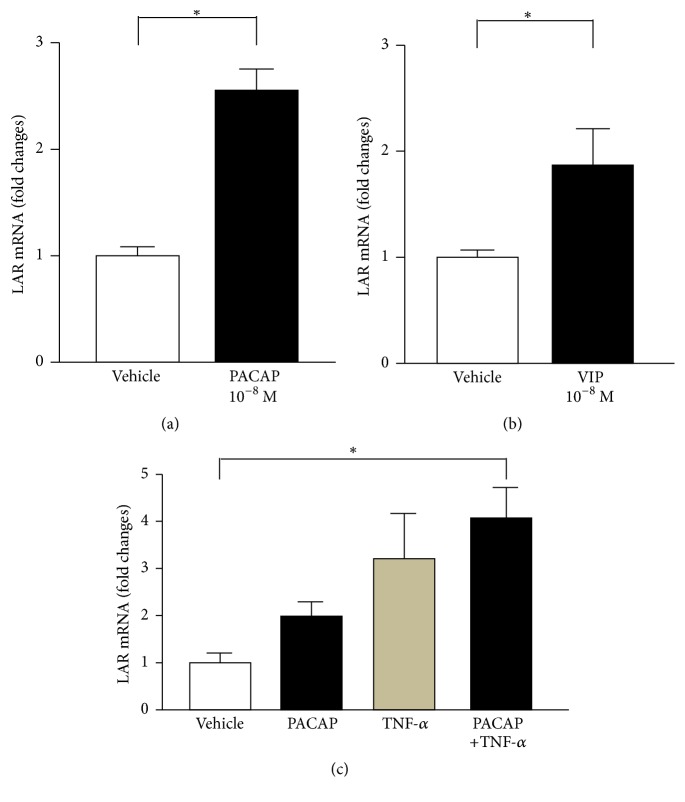
LAR expression in liver slice. (a) and (b) LAR mRNA expression in liver slices cultured with 10^−8^ M PACAP (a) or 10^−8^ M VIP (b) for 24 h. *n* = 4 for each group. (c) LAR mRNA expression in liver slices cultured with 10^−9^ M PACAP and/or 10^−10^ M TNF-*α* for 24 h. *n* = 3 for each group. Each bar represents mean ± s.e.m. ^*∗*^ *p* < 0.05.

This finding prompted us to examine direct effect of PACAP on LAR mRNA expression in the liver slices in vitro experiments. In the liver slices prepared from ICR mice and cultured in DMEM solution for 24 h, the treatment with 10^−8^ M PACAP38 markedly increased LAR mRNA expression (Figure 5(a)). VIP also significantly increased LAR mRNA expression (Figure 5(b)). Both 10^−9^ M PACAP and 10^−10^ M TNF-*α* did not increase but coadministration significantly increased LAR expression (Figure 5(c)). These results suggest that PACAP directly interacts with the liver to induce LAR mRNA expression.

### 3.3. VPAC1-R mRNA Expression Was Increased in Liver of Mice Fed HFD

**Figure 6:**
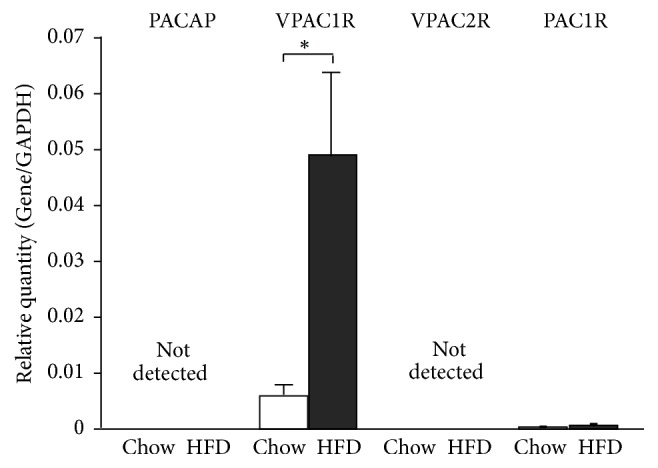
Expressions of PACAP and PACAR receptor subclasses in liver. The mRNA expressions of PACAP and PACAR receptors, VPAC1-R, VPAC2-R, and PAC1-R, in the liver of ICR mice fed regular chow and HFD for 6 weeks. *n* = 5-6 for each group. Each bar represents mean ± s.e.m. ^*∗*^ *p* < 0.05.

The mRNA expressions of PACAP and three subclasses of PACAP receptors were examined. VAPC1-R at high levels and PAC1-R at low levels were expressed, while PACAP and VPAC2-R were not expressed, in the liver (Figure 6). The mRNA expression of VAPC1-R in the liver was 10-fold elevated by HFD. These results suggest that under HFD conditions, the remarkable elevation of VPAC1-R may allow PACAP to induce LAR mRNA expression in the liver to induce insulin resistance.

## 4. Discussion

The present study aimed to clarify the mechanism underlying the antidiabetic phenotype of PACAP null mice. We found that HFD markedly increased LAR and VPAC1-R expressions in the liver, in parallel with systemic insulin resistance. Deficiency of PACAP counteracted the HFD-induced insulin resistance and elevation of LAR expression in the liver. Conversely, administration of PACAP increased LAR expression in the liver slices in vitro. The present study indicates that, under HFD conditions, the remarkable elevation of VPAC1-R may allow PACAP to induce LAR mRNA expression in the liver to induce insulin resistance.

In the present study, HFD-induced LAR expression in parallel with insulin resistance in the liver, while silencing of LAR expression in liver of HFD mice restored phosphorylation of insulin receptor. The activation of insulin receptor is triggered by its phosphorylation at tyrosine residues in response to insulin and terminated by dephosphorylation induced by protein tyrosine phosphatases (PTPs) [21–23]. Among PTPs, LAR is widely expressed in insulin-sensitive tissues and suppresses the insulin signaling in skeletal muscle and adipocyte [24, 25]. Moreover, deficiency of LAR upregulates PI3K, an activator of Akt, in hepatoma cells [26]. Furthermore, LAR expression was elevated in liver of obese Zucker fatty rat [27]. Our results, combined with these reports, suggest that the elevated LAR in liver by HFD causes insulin resistance.

Chronic inflammation plays a key role in the pathogenesis of insulin resistance [28]. TNF-*α* promotes inflammation and suppresses insulin sensitivity in insulin target tissues including liver. TNF-*α* also increases LAR expression [27]. In our previous study, while plasma TNF-*α* level of PACAP null mice was not significantly different from that of wild mice, the LAR expression in liver was reduced in PACAP null mice, showing an apparent discrepancy [14]. We here demonstrated that PACAP potentiates the TNF-*α*-induced increase in LAR expression. It is suggested that the potentiation of LAR expression by PACAP was abolished in PACAP null mice, resulting in counteraction of the insulin resistance.

In this study, PACAP was not detected in liver. Hence, the PACAP that interacts with VPAC1-R to elevate LAR may be derived from extraliver tissues. It was reported that PACAP is produced in both preganglionic and postganglionic sympathetic nerves and that PACAP-containing nerve fibers are observed in the gastrointestinal tract, adrenals, and blood vessel walls in several organs [29–32]. Hence, PACAP could be derived from sympathetic nerves innervating the liver. It should also be noted that PACAP stimulates release of glucagon and catecholamines, the agents that stimulate adenylate cyclase activity in hepatocytes [33, 34]. Hence, PACAP could stimulate glucose output from hepatocytes not only via its direct action on the liver but also via releasing glucagon and/or catecholamine [15, 16].

The inhibitor of dipeptidyl peptidase IV (DPP-IV) is clinically used to treat type 2 diabetic patients. DPP-IV inhibitors lengthen the lifetime and hence promote the effect of antidiabetic hormones, glucagon-like peptide (GLP-1), and glucose-dependent insulinotropic polypeptide (GIP). A big advantage of DPP-IV is that they induce pathologic hypoglycemia in a much smaller incidence than the classical antidiabetic drugs such as sulfonylureas [35]. The underlying mechanisms are not fully understood, though the glucose-dependent insulinotropic effects of GLP-1 and GIP are implicated. DPP-IV plays a major role in the degradation of circulating PACAP38 [36, 37]. Hence, DPP-IV inhibitors are expected to lengthen the lifetime and promote the effect of PACAP. The present study suggests a possible mechanism that the promoted action of PACAP by DPP-IV inhibitors enhances glucose output from the liver to prevent hypoglycemia.

Several reports suggest a possible involvement of PACAP in development of type 2 diabetes, although this needs to be explored more in detail. PACAP in the subpicomolar range amplifies glucose-induced insulin release from islets [1, 2], an action partly mediated by VPAC2-R [35]. VPAC2-R is located in the islets and adipocytes, and VPAC2-R specific agonists reportedly enhance insulin secretion and glucose disposal [38, 39]. In addition, the present study found that VPAC1-R in the liver is elevated and mediates insulin resistance in mice fed HFD. Collectively, the antagonism of VPAC1-R and agonism of VPAC2-R could work in concert to effectively counteract hyperglycemia [40]. Development of the selective VPAC1-R antagonist may provide a novel tool to treat diabetes and metabolic syndrome.

## Supplementary Material


**Supplementary Table 1, related to RT-PCR procedures.**Forward and reverse primers used in RT-PCR experiments.

## Abbreviations

PACAP: Pituitary adenylate cyclase-activating polypeptide
VIP: Vasoactive intestinal peptide
HFD: High-fat diet
NCD: Normal chow diet
LAR: Leukocyte common antigen-related
PTPRF: Receptor type protein tyrosine phosphatase F
GTT: Glucose tolerance test
ITT: Insulin tolerance test.
